# Ghrelin Protects Human Lens Epithelial Cells against Oxidative Stress-Induced Damage

**DOI:** 10.1155/2017/1910450

**Published:** 2017-10-10

**Authors:** Jie Bai, Fan Yang, Li Dong, Yi Zheng

**Affiliations:** ^1^Key Laboratory of Harbin Medical University Eye Center, Eye Hospital, First Affiliated Hospital, Harbin Medical University, Harbin, China; ^2^Public Department, Second Affiliated Hospital, Harbin Medical University, Harbin, China

## Abstract

Oxidative stress has been recognized as an important mediator in the pathogenesis of age-related cataracts; using antioxidant supplements is one plausible strategy to protect the antioxidative defense system against oxidative stress. Ghrelin administration is expected to reduce ROS, preventing the onset of different diseases. The role of ghrelin, if any, in protecting against oxidative stress in HLECs has never been examined. In the present study, we investigated the effects of ghrelin against H_2_O_2_-induced oxidative stress and the associated molecular mechanisms in HLECs and rat lenses. The results showed that pretreatment with ghrelin reduced H_2_O_2_-induced cellular apoptosis and ROS accumulation, increased the expression levels of SOD and CAT, and decreased the expression level of MDA. The morphological examination showed that the ghrelin-treated lens organ culture maintained transparency. This is the first report to show that ghrelin can protect HLECs from H_2_O_2_-induced oxidative stress. Our findings suggest that ghrelin may prevent the progression of cataracts, which has treatment value for ophthalmologists.

## 1. Introduction

Oxidative stress has been recognized as an important mediator in the pathogenesis of age-related cataracts; it is widely recognized as a state of imbalance between prooxidants and antioxidants [1, 2]. Excessive production of reactive oxygen species (ROS) plays an important role in the destruction of normal lens epithelial cell function [3, 4]. Hydrogen peroxide (H_2_O_2_), a nonradical member of the active oxygen family, is a major intracellular ROS and accumulates in substantial amounts in the lens [5]. Several studies have shown that free radicals and ROS can affect the growth and function of human lens epithelial cells (HLECs) [1, 2, 5]. Our previous studies have found that ROS produced by H_2_O_2_ causes protein degradation and epithelial cell damage—the damage similar to the damage found in human cataracts [6, 7].

Cataracts, which are the leading cause of visual disability globally, are a protein conformational disease characterized by the aggregation of oxidatively damaged proteins [8]. Oxidation of lens proteins is a major risk factor in cataract formation—any external insult or insufficient degradation of damaged proteins may affect the antioxidant status of the lens and cause opacification [1, 2, 7].

Using antioxidant supplements is one plausible strategy to protect the antioxidative defense system against oxidative stress [9]. Although surgery can remedy cataracts, there are numerous postoperative complications; further, some regions lack the necessary surgical instruments and face shortages of doctors who can meet the medical needs of large numbers of patients. These factors limit the availability of cataract surgery. There are no effective therapeutic agents to halt the formation of a cataractous lens; therefore, it is necessary to develop a pharmacological intervention to improve lens transparency and delay the progression of cataracts.

The growth hormone-releasing peptide ghrelin, a 28-amino-acid endogenous peptide, is secreted primarily from the gastric mucosa [10]. Its transcripts have also been found in the intestine, pancreas, liver, placenta, heart, lungs, central nervous system (CNS), and kidneys, suggesting its extraendocrine as well as endocrine action. Studies have illustrated that ghrelin exhibits favorable cytoprotective effects against oxidative stress [11]. It can remove ROS and reactive nitrogen species (RNSs) by increasing the expression of antioxidant enzymes and directly scavenging free radicals [12]. Ghrelin administration is expected to reduce ROS, preventing the onset of various diseases. The role of ghrelin, if any, in protecting against oxidative stress in HLECs has never been examined. In the present study, we investigated the effects of ghrelin against H_2_O_2_-induced oxidative stress and the associated molecular mechanisms in HLECs and rat lenses.

## 2. Material and Methods

### 2.1. Reagents and Antibodies

Ghrelin was purchased from Sigma Chemical (St. Louis, MO, USA) and dissolved in dimethylsulfoxide (DMSO) (Sigma, St. Louis, MO). Fetal bovine serum (FBS) and Dulbecco's modified Eagle's medium (DMEM) were obtained from Gibco (Grand Island, NY, USA). Medium 199 was obtained from Sigma-Aldrich (St. Louis, MO). Annexin V-FITC and propidium iodide (PI) were obtained from Becton Dickinson (Mountain View, CA, USA). 3-(4,5-dimethyl-2-thiazolyl)-2,5-diphenyl-2H-tetrazolium bromide (MTT) and H_2_DCFDA were obtained from Beyotime (Beyotime Institute of Biotechnology, Shanghai, China). Anti-SOD, anti-CAT, and anti-MDA antibodies were purchased from Santa Cruz Biotechnology Inc. (Santa Cruz, CA, USA). SOD, CAT, and MDA colorimetric kits were purchased from Nanjing Jiancheng Bioengineering Institute (Nanjing, China).

### 2.2. Cell Culture and Treatment

HLECs (ATCC, America) were cultured in DMEM with heat-inactivated (56°C, 0.5 h) FBS (15%), 100 U/mL penicillin, and 100 mg/mL streptomycin in humidified 5% CO_2_ at 37°C. The cells were routinely subcultured every 2-3 days. When grown to 70% confluence, they were treated with the indicated concentration of H_2_O_2_ for 24 h or pretreated with different concentrations of ghrelin for 12 h before the H_2_O_2_ treatment.

### 2.3. Cell Viability Assay

The cells were plated at a density of 2 × 10^4^ cells/well in a 96-well culture plate and incubated with 0, 50, 100, 200, 400, and 800 *μ*M H_2_O_2_ for 24 h alone or after pretreatment with different concentrations of ghrelin (10^−9^–10^−6^ M) for 12 h. The culture medium was removed, and cell viability was measured using the MTT method as previously described [13].

The morphological changes of the cells were observed under an inverted microscope (Olympus CK-30, Tokyo, Japan).

### 2.4. Cell Apoptosis Assay

Annexin V-FITC/PI staining was used to quantify the amount of cell apoptosis. Cells were plated and incubated on a six-well plate at 1 × 10^6^ cells/well and pretreated with or without different concentrations of ghrelin for 12 h, after which they were treated with 100 *μ*M H_2_O_2_ for 24 h. The cells were collected and stained with annexin V-FITC/PI in binding buffer at room temperature in the dark for 20 min. The stained cells were then analyzed using a flow cytometry system.

### 2.5. Fluorescent Staining of Cells with H_2_DCFDA

The levels of intracellular ROS were monitored using H_2_DCFDA. The cells were incubated in 10 *μ*M H_2_DCFDA for 20 min at 37°C and then washed twice with PBS. The fluorescence intensity of DCF was detected using a fluorescence microscope (Leica DMI 4000, Germany).

### 2.6. Lens Organ Culture

Rat eyes (Harbin Medical University, Harbin, China) were removed and placed in mammalian physiological saline prewarmed to 37°C. Freshly extracted transparent lenses were incubated in Medium 199 containing 50 mg/mL gentamicin and 0.1% BSA with 5% CO_2_ at 37°C. The lenses were treated with ghrelin at concentrations of 10^−9^–10^−6^ M for 24 h at 37°C. The medium was changed, and 100 *μ*M H_2_O_2_ was added to the medium for 6 h. The lenses were observed under a stereomicroscope and photographed against a background of black gridlines to record the development of opacity.

### 2.7. Antioxidant Enzyme Content Assay

Whole lenses were removed from the eyes and sonicated in extract buffer. After centrifugation, the supernatants were used for testing according to the assay manufacturer's instructions. Total SOD content was determined spectrophotometrically at 550 nm, and the results were expressed as U·mg^−1^ protein [14]. MDA was measured with reference to the MDA assay kit using the thiobarbituric acid method [15]. The results were expressed as U·mg^−1^, and the intensity of the resulting pink color was read at 532 nm. CAT content was assayed using the ammonium molybdate method according to the instructions of the CAT assay kit [16]. The results were expressed as U·mg^−1^, and the faint yellow complexes were detected at 405 nm.

### 2.8. Western Blot Analysis

Lenses from each group were washed with cold saline, dried with filter paper, cut with Vannas scissors, and then ground on ice. The tissue was spun down in a refrigerated centrifuge at 1000 r/min for 10 min, and the supernatants were lysed on ice for 20 min in radioimmunoprecipitation assay (RIPA) buffer containing a protease inhibitor cocktail and centrifuged at 12000*g* for 20 min. The total protein (30 *μ*g) was subjected to 10–15% SDS-PAGE and transferred into a polyvinylidene difluoride membrane. The blot was incubated with antibodies against SOD, CAT, and MDA. The enzyme was used with a horseradish peroxidase-conjugated secondary antibody. Enhanced chemiluminescence was used to detect the immunoreactive bands, and ImageJ software (image processing and analysis in Java) was used to quantify the results. After normalizing to the individual actine levels, the ratio of the expression of target proteins was determined. Each experiment was repeated three times.

### 2.9. Statistical Analysis

Statistical analysis was carried out using GraphPad Prism 5.0 (GraphPad Software, San Diego, CA). All values are expressed as the mean ± standard error of the mean (SEM) from at least three independent experiments. The significance of pairwise group was evaluated using Student's *t*-test. For comparison of more than two groups, one-way ANOVA was used; *P* < 0.05 was considered to be significant.

## 3. Results

### 3.1. Effects of H_2_O_2_ and Ghrelin on HLECs' Viability

**Figure 1:**
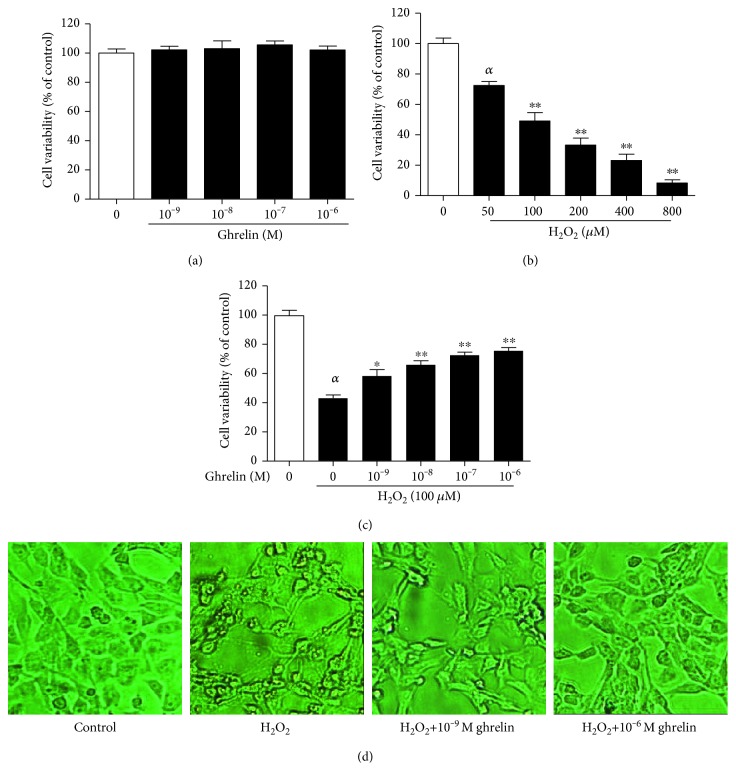
The effects of ghrelin on the cell viability of H_2_O_2_-treated HLECs. (a) HLECs were incubated with different concentrations of ghrelin (10^−9^–10^−6^ M) for 24 h. (b) HLECs were incubated with different concentrations of H_2_O_2_ (50–800 *μ*M) for 24 h. (c) HLECs were preincubated with ghrelin (10^−9^–10^−6^ M) for 12 h before being treated with 100 *μ*M H_2_O_2_ for 24 h. Cell viability was assessed via MTT assay. (d) HLECs were detected by microscopy. The results were represented as the mean ± SEM (*n* = 3) from three independent experiments. *^α^P* < 0.01, compared with the untreated control group; ^∗^*P* < 0.05, ^∗∗^*P* < 0.01, compared with the H_2_O_2_-treated group.

As shown in Figure 1(a), ghrelin did not exhibit any cytotoxic effects on HLECs. H_2_O_2_ impaired cell viability in a dose-dependent manner (Figure 1(b)). Treatment with a concentration of 100 *μ*Μ H_2_O_2_ for 24 h was selected for subsequent experiments because it reduced cell viability to approximately 49.12% compared with the control group (cells not treated with H_2_O_2_). The pretreatment of HLECs with ghrelin showed a dose-dependent protective effect against H_2_O_2_ damage (Figure 1(c)).  Figure 1(d) shows the morphological changes of HLECs. The control group showed normal cell morphology and a regular range of cells with intact junctions. The H_2_O_2_-treated group showed shrinkage of the cells with abnormal shapes, and the distance between cells increased; however, pretreatment with ghrelin inhibited H_2_O_2_ damage to cell morphology.

### 3.2. Ghrelin Inhibits Apoptosis Induced by H_2_O_2_

**Figure 2:**
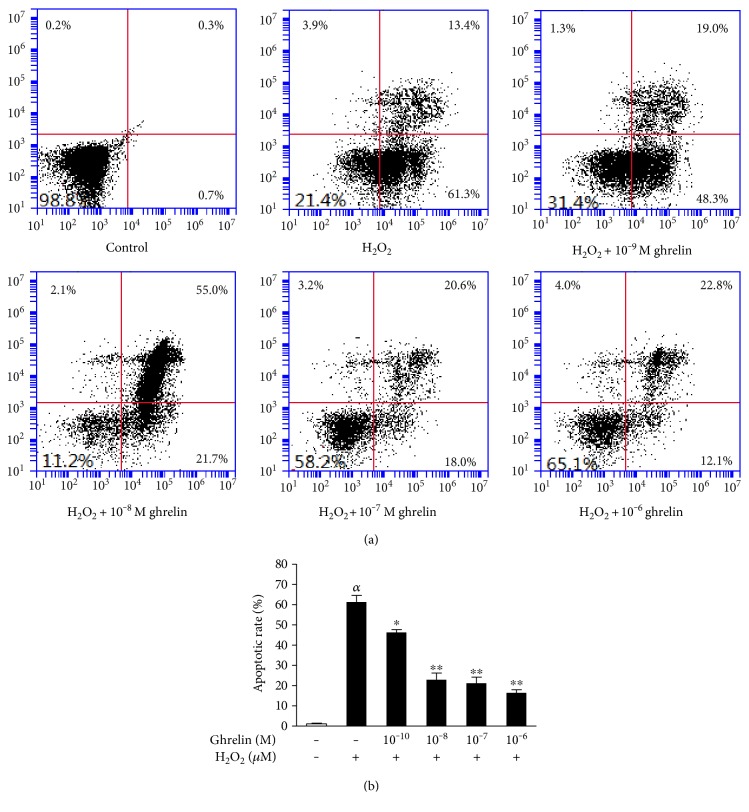
Ghrelin prevented H_2_O_2_-induced cellular apoptosis in HLECs. HLECs were preincubated with ghrelin (10^−9^–10^−6^ M) for 12 h before being treated with 100 *μ*M H_2_O_2_ for 24 h. (a) Apoptosis of HLECs as detected by flow cytometry. The results were represented as the mean ± SEM (*n* = 3) from three independent experiments. (b) Ghrelin significantly decreased the apoptosis rate of HLECs. *^α^P* < 0.01, compared with the untreated control group; ^∗^*P* < 0.05, ^∗∗^*P* < 0.01, compared with the H_2_O_2_-treated group.

The rate of cell apoptosis was quantified using flow cytometric analysis by double staining with annexin V-FITC and PI. An increase of apoptotic cells was observed in the H_2_O_2_-treated group, while ghrelin pretreatment decreased the apoptosis rate of the cells exposed to H_2_O_2_ (Figure 2(a)). This positive effect of ghrelin was observed in a concentration-dependent manner (Figure 2(b)).

### 3.3. Ghrelin Reduced the Generation of ROS in HLECs

**Figure 3:**
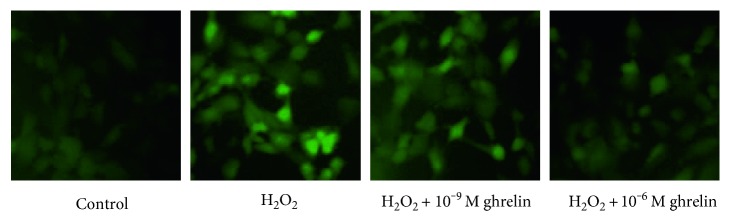
Effect of ghrelin on H_2_O_2_-induced generation of ROS in HLECs. Cells were treated with 100 *μ*M H_2_O_2_ for 24 h after incubation in the absence or presence of ghrelin for 12 h. Then, the production of ROS was determined using 10 *μ*M H_2_DCFDA. The morphological features of the cells were observed using fluorescence microscopy.

As expected, there was a lack of staining in the H_2_O_2_-free control group (Figure 3). HLECs that were only exposed to H_2_O_2_ had a light green color, indicating that there was a marked increase in ROS levels. This increase in intracellular ROS was prevented in a dose-dependent manner by pretreatment with ghrelin. This finding indicates that ghrelin can prevent the generation of intracellular ROS in HLECs challenged with H_2_O_2_.

### 3.4. Grading the Lenses

**Figure 4:**
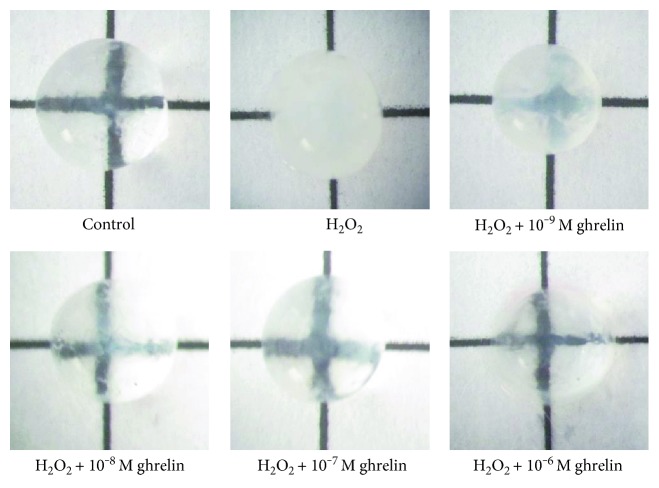
Morphological observations confirmed the reduction in opacity when the lenses were incubated in ghrelin. Whole lenses were pretreated with ghrelin at concentrations of 10^−9^–10^−6^ M for 24 h and then cultured for 6 h with 100 *μ*M H_2_O_2_. The control group received no treatment, and the H_2_O_2_ group was treated only with 100 *μ*M H_2_O. Photographs were taken against a background of black gridlines. The higher the concentrations of ghrelin, the less opacity was observed in the lenses.

We developed an organ culture experiment to examine the effects of ghrelin on the lenses. Morphological observations confirmed that the lenses incubated in ghrelin showed a reduction in opacity (Figure 4). Lenses in the control group had an absence of opacification, and the gridlines were clearly visible; however, lens opacity measurements showed that 100 *μ*M H_2_O_2_ induced obvious cataract formation in the lenses. Ghrelin blocked the effect of H_2_O_2_ in a dose-dependent manner.

### 3.5. Effects of Ghrelin on the Protein Expression of SOD, CAT, and MDA in Lenses

**Figure 5:**
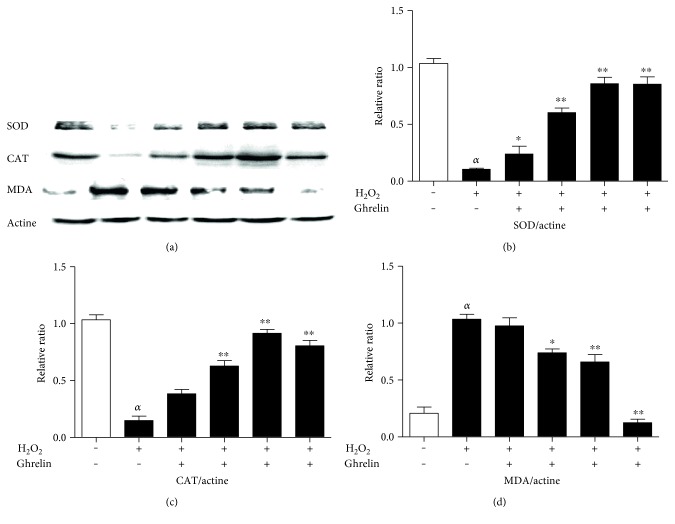
Effects of ghrelin on expression of SOD, CAT, and MDA in lenses. (a) The protein levels of SOD, CAT, and MDA were measured using western blot analyses. (b, c, d) The relative protein expression levels of SOD, CAT, and MDA. *^α^P* < 0.01, compared with the untreated control group; ^∗^*P* < 0.05, ^∗∗^*P* < 0.01, compared with the H_2_O_2_-treated group.

Western blot was used to measure the levels of SOD, CAT, and MDA in H_2_O_2_-stimulated HLECs to detect the antioxidative capability of ghrelin. Exposure to H_2_O_2_ markedly decreased the activity of SOD and CAT and increased the MDA content compared with the control group. Pretreatment with ghrelin significantly increased the expression of SOD and CAT, and decreased MDA compared with the H_2_O_2_ group (Figure 5).

### 3.6. Effect of Ghrelin on SOD, CAT Activity, and MDA Content in Lenses

The biological activity assay showed that 100 mM H_2_O_2_ significantly decreased SOD and CAT activity in the lenses, and pretreatment with ghrelin blocked the effect of H_2_O_2_. As shown in Table 1, 100 mM H_2_O_2_ significantly increased MDA content in the lenses. Finally, ghrelin blocked the effect of H_2_O_2_ in a dose-dependent manner.

## 4. Discussion and Conclusion

Cataracts are the leading cause of legal blindness worldwide. Until now, there has not been an effective pharmacological agent that can inhibit or reverse the progression of cataracts, and the search for affordable and nonsurgical pharmacological treatment is necessary to delay the progression of lens opacification [17, 18]. The effects of ghrelin on HLECs and the regulatory mechanism of those effects have never been reported. Therefore, the present study is the first to investigate the effect of ghrelin on H_2_O_2_-induced cell injury in HLECs.

Accumulating evidence shows that oxidative stress is a major contributor to the initiation and progression of cataracts. Oxidative stress refers to a state of elevated levels of ROS, with the latter being affected by an intracellular oxidative and antioxidant defense mechanism [19–21]. An increasing level of ROS is a crucial determinant of oxidative damage and impaired cellular function. During oxidative stress, the activity of antioxidant enzymes is inhibited, while the concentration of reactive nitrogen species (RNSs) or ROS increases dramatically. Oxidative stress is involved in the pathogenesis of eye diseases, including age-related cataracts and macular degeneration [19, 22]. Considering that oxidative stress plays an important role in the pathogenesis of cataracts, reducing oxidative stress is a plausible potential therapeutic target for cataracts.

Ghrelin has several biological actions, including regulating cell survival and proliferation, inhibiting inflammation, and exerting antioxidative effects [23, 24]. Previous studies have indicated that the antioxidative effect of ghrelin is based on increasing the activity of endogenous antioxidant enzymes [25]. For example, ghrelin has been reported to alleviate SAH-induced oxidative brain damage by maintaining a balance in oxidant-antioxidant status [26]. Ghrelin has also been found to significantly reduce the protein expression of iNOS and increase the expression of CuZnSOD, MnSOD, CAT, and GPx in the liver [27]. Ghrelin is an amino acid peptide—it is safe for humans, as has been shown in many studies [28]. Our study also showed that ghrelin exhibited no obvious cytotoxicity in HLECs and pretreatment with ghrelin inhibited H_2_O_2_ damage to cell morphology. Considering its lack of toxicity and its excellent antioxidative effect, we propose that ghrelin should be considered as a prophylaxis for preserving visual function and could be used to treat age-related cataracts.

HLECs are a single layer of epithelial cells on the lens' anterior surface. The normal construction and function of HLECs is crucial for the maintenance of the transparency and metabolic homeostasis of the entire lens. Once damaged, they cannot self-renew, and they become permanently impaired. Oxidative stress could increase the permeability of HLECs, causing dysfunction. Studies have indicated that oxidants, especially H_2_O_2_, could trigger lens epithelial cell apoptosis and initiate early cataract formation [6, 7, 29]. Once the lens' defense system is weakened, cataracts begin to form.

The antioxidant systems protect cells against oxidative damage during normal metabolism and after an oxidative insult; they contain numerous antioxidant enzymes such as SOD, CAT, and glutathione peroxidase (GPx). These antioxidative enzymes can protect the lens from oxidative stress and maintain lens clarity [30]. H_2_O_2_-induced cataracts were associated with decreases in SOD and CAT activity and increase in MDA activity in the lens. These three enzymes are essential in oxidative stress protection and normal lens metabolism (SOD, CAT, and MDA). The antioxidant enzymes are able to catalytically remove free radicals and other reactive species. SOD can prevent lipid peroxidation, scavenge ROS, and protect cells from the damaging effects of toxic oxygen radicals [31]. CAT reduces H_2_O_2_ to water; a decline in the level of CAT weakens the antioxidant capacity of the lens epithelial cells and induces their apoptosis, which causes cataracts [32]. MDA is one of the metabolic products of lipid peroxides (LPO) and is well known as a widely used marker for oxidative stress [33].

Our in vitro test has verified the effect of ghrelin in preventing oxidative stress-induced cell dysfunction; however, can ghrelin also be effective for lens tissue? To answer this, we present the results of an ex vivo study to assess the anticataract potential of ghrelin in H_2_O_2_-induced isolated rat lenses through observation of lens transparency and estimation of some biochemical parameters such as SOD, CAT, and MDA contents.

Western blot results showed increased protein expression of SOD and CAT and a decreased expression of MDA under ghrelin treatment. Antioxidant enzyme content assays showed similar results; the mean content of SOD and CAT significantly decreased in the H_2_O_2_-treated lenses compared with the control lenses. In the ghrelin-treated group, the mean content of antioxidant enzymes was restored compared with the lenses in the H_2_O_2_-treated group. The elevated content of MDA in the H_2_O_2_-treated lenses may account for the disruption of membrane lipids. In addition, the reduction of the MDA level in ghrelin-treated group suggests that ghrelin may have prevented the disruption of lenticular membrane lipids, thereby impeding opacification of the lens.

In conclusion, the data from our experiments demonstrated that ghrelin effectively retarded H_2_O_2_-induced cataract formation. As an antioxidant agent, ghrelin increased the levels of SOD and CAT and decreased the level of MDA, thus sustaining lens transparency. Furthermore, ghrelin is an amino acid and should therefore be safe for use in humans. Due to its safety and efficacy, ghrelin has potentially important implications for the prevention of cataractogenesis.

## Figures and Tables

**Table 1:** Effect of ghrelin on SOD, CAT activity, and MDA content in lenses.

Group	SOD (U·mg^−1^)	CAT (U·mg^−1^)	MDA (nmol·mg^−1^)
Control	27.87 ± 1.57	7.47 ± 1.43	0.44 ± 0.02
H_2_O_2_	10.27 ± 1.03^a^	3.34 ± 1.15^a^	1.83 ± 0.09^a^
H_2_O_2_ + 10^−9^ M ghrelin	15.29 ± 2.11^b^	4.99 ± 1.23^b^	0.93 ± 0.04^b^
H_2_O_2_ + 10^−8^ M ghrelin	15.43 ± 2.00^b^	4.87 ± 1.01^b^	0.86 ± 0.05^b^
H_2_O_2_ + 10^−7^ M ghrelin	17.03 ± 1.59^b^	5.34 ± 0.74^b^	0.77 ± 0.04^b^
H_2_O_2_ + 10^−6^ M ghrelin	19.03 ± 2.08^b^	5.65 ± 0.81^b^	0.67 ± 0.07^b^

Compared with control group ^a^*P* < 0.05; compared with the H_2_O_2_-treated group ^b^*P* < 0.05.

## Abbreviations

ROS: Reactive oxygen species
HLECs: Human lens epithelial cells
H_2_O_2:_: Hydrogen peroxide
SOD: Superoxide dismutase
CAT: Catalase
MDA: Malondialdehyde
MTT: 3-(4,5-Dimethyl-2-thiazolyl)-2,5-diphenyl-2H-tetrazolium bromide.
